# Cell Therapies for Heart Function Recovery: Focus on Myocardial Tissue Engineering and Nanotechnologies

**DOI:** 10.1155/2012/971614

**Published:** 2012-04-22

**Authors:** Marie-Noëlle Giraud, Anne Géraldine Guex, Hendrik T. Tevaearai

**Affiliations:** ^1^Cardiology, Department of Medicine, Faculty of Science, University of Fribourg, Chemin du Musée 5, 1700 Fribourg, Switzerland; ^2^Clinic for Cardiovascular Surgery, Inselspital Berne, Berne University Hospital and University of Berne, Switzerland; ^3^Empa, Swiss Federal Laboratories for Material Science and Technology, 9014 St. Gallen, Switzerland

## Abstract

Cell therapies have gained increasing interest and developed in several approaches related to the treatment of damaged myocardium. The results of multiple clinical trials have already been reported, almost exclusively involving the direct injection of stem cells. It has, however, been postulated that the efficiency of injected cells could possibly be hindered by the mechanical trauma due to the injection and their low survival in the hostile environment. It has indeed been demonstrated that cell mortality due to the injection approaches 90%. Major issues still need to be resolved and bed-to-bench followup is paramount to foster clinical implementations. The tissue engineering approach thus constitutes an attractive alternative since it provides the opportunity to deliver a large number of cells that are already organized in an extracellular matrix. Recent laboratory reports confirmed the interest of this approach and already encouraged a few groups to investigate it in clinical studies. We discuss current knowledge regarding engineered tissue for myocardial repair or replacement and in particular the recent implementation of nanotechnological approaches.

## 1. Introduction

It was long believed that the adult heart does not regenerate. The recent discovery of cardiac stem cells (CSCs), however, challenged this dogma [1]. Since then, several populations of CSCs have been identified and distinguished by means of their surface markers. In addition, using a fascinating approach based on the comparison of C14 incorporation before and after the explosion of the atomic bomb, Bergmann et al. recently demonstrated that human cardio-myocytes in fact regenerate at a rate of approximately one percent per year at the age of 25 and 0.45% at the age of 75 [2]. 

**Figure 1:**
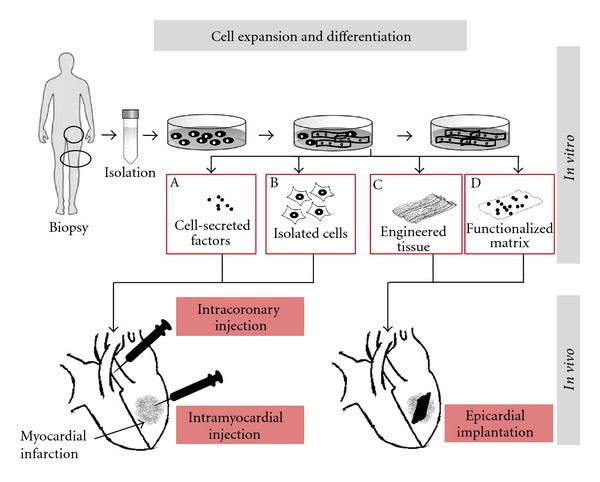
Cell therapy approaches for myocardial infarction: cells are isolated from biopsies, expanded, and eventually differentiated *in vitro* following specific culture conditions. Conditioned medium containing secreted or lyophilized factors (A) or isolated cells (B) are injected directly within the myocardium or within the coronaries. Further *in vitro* process from cultured cells enables the development of structured engineered muscle tissue with or without contracting properties that can be directly sutured or glued at the surface of the infarct (C). Functionalized matrix combining biologically active factors and engrafted cells (D) represents a more sophisticated alternative.

Beside their possible implication in this regenerative process, the exact physiological function of CSCs has not yet been fully clarified. Their role in pathological situations is also unclear since, in case of myocardial injury such as after a myocardial infarction, their potential regenerative capacity is clearly overwhelmed. Nevertheless, the rapid progress in understanding myocardial regenerative mechanisms continues to encourage the scientific and clinical communities to multiply the laboratory investigations and consider the value of stem cell therapy in clinical protocols. Depending on the clinical need and the rationale, transplantation of isolated cells or implantation of an engineered muscle graft is under consideration as presented in Figure 1. As illustrated, the concept for cell-based therapy is thus quite straightforward; however, its implementation faces numerous challenges.

In this paper we present the important questions that remain to be investigated to ascertain a successful translation of current experimental knowledge regarding cell therapy for myocardial repair/replacement. In particular, we emphasize the critical importance of favoring a multidisciplinary approach including biotechnologies, material science, and nanotechnologies to engineer myocardial tissue.

## 2. Clinical Trials

Compelling evidence of the beneficial effect of isolated cell transplantation to the heart including improvement in cardiac contractile function, decrease in left ventricular remodeling, reduction of the infarct size, and increase in vascular density was provided by early experimental studies [3]. Consequently, rapid clinical trials testing the safety and efficiency of cell therapy have been undertaken and are ongoing [4–6]. However, in a general manner, beneficial effects on heart function and regeneration observed after cell therapy in animal models were not always followed by convincing clinical outcomes [7, 8]. The modest or absent improvement of heart function has been confirmed in the Cochrane report, presenting a recent meta-analysis focusing on bone marrow stem cells transplantation [9]. It has been hypothesized that the modest or indeed lack of functional improvement may be the result of poor cell specificity and quality as well as technical pitfalls during injection. The report concluded with the following major issue to be investigated: define the optimal type and the dose of stem cells, the route and timing of delivery after myocardial infarction, and long-term outcomes. In addition, mechanisms of action and in particular the role of injected stem cells in the management of acute myocardial infarction are of particular relevance to improve treatment efficiency. Furthermore, the possibility that the injured microenvironment has low ability to permit cell survival and differentiation has been raised. Indeed, the rather hostile, hypoxic, stressed and remodeled cardiac environment as well as the immunologic and inflammatory milieu related to the patient's disease is certainly unfavorable conditions for cell growth and differentiation. 

The search for new strategies to overcome drawbacks from direct cell implantation has resulted in an increased interest for myocardial tissue engineering. Recent studies provide convincing experimental short-term outcomes showing recovery of heart function; our group contributed to this proof of concept with several types of engineered tissues investigated for functional recovery including long-term followup [10–12]. To date, the first two clinical trials have been initiated [13, 14]. The first twenty patients with postinfarction myocardial scar received autologous bone marrow stem cells either directly injected in and around the infarct or seeded on a collagen matrix, which was then placed and sutured on the infarcted area. This pioneer study not only confirmed the feasibility and safety of the procedure but also already suggested a benefit in favor of the combination of cells and matrix. Results of the recently launched second clinical trial, describing the implantation of an engineered construct composed of stacks of myoblast sheets, are pending [14].

## 3. Major Challenges: What Research Is Needed to Make Cell-Based Treatments a Reality?

### 3.1. *In Vivo* Investigations

The importance of experimental settings and in particular large animal models to provide predictor features for cell therapy applied in human clinical trials has been emphasized by van der Spoel et al. [15]. The authors performed a meta-analysis for cell therapy on large animal models of acute and chronic cardiac ischemia. They determined a short-term 7.5% global ejection function improvement due to an increased end systolic volume. They reported a prevalence of mesenchymal stem cells (MSCs), a high number of cells, and better outcomes for chronic ischemia. However, no effect of distinct delivery routes was examined.

Experimental and preclinical investigations have mostly been performed in small (rodent) or large (pig) animal models of heart failure following a myocardial infarction induced by ligation of the left anterior descending coronary artery (LAD ligation). Various treatments have been tested and compared (Figure 1): cells were applied in acute or chronic phases, cells were injected into the scar or at its periphery, and tissue constructs were glued or sutured at the surface of the infarcted area. Typically, morphological and functional changes of the treated hearts were followed by repeated echocardiography or MRI and generally over a 4-week follow-up period [10, 16, 17]. At the end of this observation period and before sacrifice, additional invasive investigations were performed using, for example, a pressure or a conductance microtip catheter to record and analyze complementary contractile parameters.

These studies allowed assessment of functional outcomes. Furthermore, increased interest in cell tracking, cell integration, and survival permitted the development and optimization of state-of-the-art technologies as described by Terrovitis et al. [18]. In addition, extensive efforts focusing on proteomics and high-throughput screening will enable the discovery of major mechanisms of action and important factors for myocardial recovery and repair [19].

### 3.2. Mechanisms of Action

Experimental cell therapy investigations showed beneficial outcomes including significant improvement in ventricular function, increased wall thickness, and decreased end diastolic and systolic volumes as well as neovascularisation of the scar area. However, decrease of the infarct size suggesting myogenesis is still a matter of debate and seems to be dependent on the type of cells that were implanted [20–22].

Several potential hypotheses have been raised to explain the effects and remain to be further investigated. First, a girding effect attenuating the adverse remodeling has been proposed, suggesting prevention of dilatation, modification of the scar elasticity, and an increase in wall thickness due to a cluster of cells or implanted tissues. This effect may have a minor impact as the large number of cells washed out after injection results in very small clusters of cells that may not be sufficient to produce the adequate mechanical strength to prevent remodeling. Furthermore, implantation of an acellular scaffold has little or no effect on cardiac function compared to the implantation of engineered tissues [12]. Second, the replacement of lost cardiomyocytes by transplanted cells is a major issue for tissue repair. Only investigations using neonatal cardiomyocytes and embryonic stem cells could report the presence of new cardiomyocytes in the periphery of implanted cells [23]. Although the delivery of embryonic stem cells or cardiac progenitor cells as committed cells to cardiac lineage could reinforce muscle contractility after differentiation and may contribute to systolic force, myogenesis is unlikely to explain the positive outcomes observed after cell therapy using other cell types.

Growing numbers of studies provide evidence that the beneficial effect of delivered cells is mediated via a paracrine effect. Cell secretions of cardioprotective, angiogenic, or stem-cell-recruiting factors are expected to trigger heart regeneration. A large panel of secreted cytokines and chemoattractants [1] are believed to bring their beneficial effect to the failing heart and suggest a multifactorial effect on angiogenesis [24], inhibition of cardiomyocyte apoptosis [25], antifibrotic effects [26], and mobilization of endogenous stem cells [1] as well modulation of the inflammatory processes [27].

### 3.3. Cell

#### 3.3.1. Source and Cell Type

Cell types and their potential for new medical treatment are presented in Tables 1 and 2. Stem cells represent a promising cell source due to their high potential for differentiation and expansion capacity. 

#### 3.3.2. How Many Cells Are Required?

Cell survival and engraftment in a hostile environment with inflammation, fibrosis, and hypoxia are a major concern. It has been demonstrated that more than 90% of injected cells are lost within the first minutes following injection. Optimization of cell retention after injection, engraftment, and survival are of paramount importance to further define the optimal quantity of cells to be implanted. So far, to overcome this effect, large numbers of cells have been injected. Dose effect has largely been reported [15]. Therefore, the injection of a high number of cells will require a high expansion capacity of autologous cells and a massive capacity of expansion if heterologous cells are used. Alternatively, strategies to improve cell engraftment and survival have been developed and include preconditioning of the cells prior to transplantation (heat shock, hypoxia), increased expression of survival factors, exposition to prosurvival factors, and the implantation of engineered tissue. 

#### 3.3.3. Further Aspects to Be Considered

Some cell-specific drawbacks are listed in Table 2. Clinical availability is one important feature to take into account in the choice of the cell source and may limit their relevance in a translational perspective. Neonatal cardiomyocytes, for example, have been widely used in preclinical studies; however, they were not exploitable for clinical studies due to low accessibility and ethical concerns. The same concerns applied for embryonic stem cells and increased research investigations to assess their teratogenicity must be undertaken before safe clinical use. Induced pluripotent stem cells (iPSCs) overcome some major shortcomings such as accessibility, expansion, and capacity; however, significant improvements to generate clinical-grade iPSCs are required for clinical translation. 

Purification is also an important feature. The selection of population clones or heterogenous population of adult stem cells has been investigated; however, it is not yet clear what type of cell population has the best regenerative capacity. Furthermore, immunogenicity of the heterologous cell may limit cell survival. Several lines of research must be carried out to identify the most efficient therapeutic candidate for patients with cardiovascular diseases. 

### 3.4. When?

The development of cardiac infarct following ischemic injury is rapidly and sequentially associated with cell death, release of paracrine factors, inflammation with leucocytes infiltration, the formation of granulation tissue composed of myofibroblast, macrophage, and collagen, spreading of the initial injury to adjacent tissue, and finally fibrosis. The reorganization of the extracellular matrix allows for compensation of the loss of cardiomyocytes. This remodeling will progressively lead to a reduction of cardiac wall thickness, ventricle dilatation, and more severe heart failure. The therapeutic target will define the cell therapy strategy. Therapeutic angiogenesis and/or myogenesis using cell or tissue transplantation might be promising therapeutic strategies in patients with severe ischemic heart disease or patients with end-stage heart failure. Alternatively, stimulation of the regenerative process may preferentially be beneficial for acute ischemia. Using a rat myocardial infarction model, Hu et al. [29] provided evidence of better outcomes when MSC were implanted 1 week after infarction. The authors suggested that reduced inflammation as well as early time point in tissue remodeling towards scar formation favors cell engraftment and angiogenesis.

### 3.5. Where?

Feasibility, safety, and cell retention are the common features that may drive the choice of the cell delivery. Different ways to inject the cells have been investigated in clinical trials, such as intracoronary (IC), intramyocardial (IM), transendocardial, interstitial retrograde coronary venous (IVR), epicardial, or systemic injection. Hou et al. [30] quantified the retention rates of peripheral blood mononuclear cells within the swine ischemic heart: IM resulted in a most efficient delivery mode with 11% of cell retention 1 hour after cell delivery but with a large variability compared to other techniques. The retention efficiency was confirmed in a rodent model after injection of cardiosphere-derived cells [31]. However, the authors also reported that IM injection can result in cell loss through the needle track and coronary venous vessels. In addition, IM is also known to induce myocardium injuries at the site of needle insertion. To date, the optimal delivery route has not been identified. Their respective advantages and disadvantages are reviewed by Dib et al. [32].

## 4. Cell Delivery through Engineered Tissue

The controllable scaffolds and culture conditions made possible by tissue engineering approaches allow the design of an adequate microenvironment that not only permits preconditioning the cells *in vitro* and provides a differentiation direction of the tissue before it is implanted for the preparation of cells prior to their implantation but also would overcome major drawbacks of isolated cells' transplantation related to their survival in inadequate ischemic tissue. In fact, the matrix of the engineered tissue can be compared to the ECM in a corresponding natural tissue. Its function during the tissue engineering process must, however, be distinguished between the *in vitro* period (preimplantation or maturation period), the surgery period (implantation period), and the *in vivo* period (postimplantation period). Regarding the *in vitro* period, the matrix could be assimilated to a biocompatible and nontoxic support material for the cells that are planned to be transplanted. The ideal matrices should thus consist of a two- or a three-dimensional structure that should favor not only cells' attachment and growth but also their further organization and possibly differentiation toward a highly ordered formation including intercellular contacts.

Considering the implantation phase, the matrix offers a significant practical advantage over the direct injection of cells. For instance, engineered myocardial biografts may be considered as a bandage that can be easily and rapidly applied at the surface of the infarcted zone. Conversely, the traditional application of cell therapy requires multiple injections to cover a similar zone. The role of the matrix during the *in vivo* phase is variable. On one hand, it may represent a structurally resistant element that can withstand the high and permanent mechanical stresses observed during the cardiac contraction/relaxation cycles. A second major role during this period is its integration within the host tissue and eventual replacement by a host ECM. For example, recent data have shown increasing evidence that the matrices may provide specific signals that will trigger the behavior of the seeded as well as the host cells. 

Various approaches have already demonstrated the possibility of designing myocardial-like structures and are detailed in recent reviews [33–36]. Briefly, one typical method consists of engineering a construct *in vitro* by combining a polymeric or a biological scaffold organized in a 3-dimensional matrix onto which cells will be seeded [20, 37, 38]. An updated list of biomaterials used for the treatment of myocardial infarction was recently assembled by Rane and Christman [39]. A second alternative takes advantage of the self-aggregation of cells when cultured in high density together with collagen, fibrin, laminin, or fibronectin [40]. In another recently described technique, the controlled recellularization of a previously decellularized natural matrix was proposed and showed spectacular results using an entire rat heart [41]. Bioprinting is also an interesting technology which essentially uses the inkjet printing principle to precisely distribute biological material within a culture's semisolid substrate [42]. Finally, an interesting approach that takes advantage of the temperature-dependent hydrophobic/hydrophilic properties of the culture dishes consists of creating monolayered cell sheets to be implanted directly at the surface of the heart [43]. Amazingly, this can be repeated so that several sheets may be stacked on top of each other in order to create a vascularized tissue of up to 1 mm thickness [44]. As opposed to the first approaches described here, the cell sheet approach does not involve the transplantation of an artificial extracellular matrix. Nevertheless, the potential of matrices may be extended since these structures can be “functionalized” through the addition of chemical compounds or proteins, which may provide specific signals that will trigger the behavior of the seeded as well as the host cells.

## 5. How Nanotechnology Will Help?

**Figure 2:**
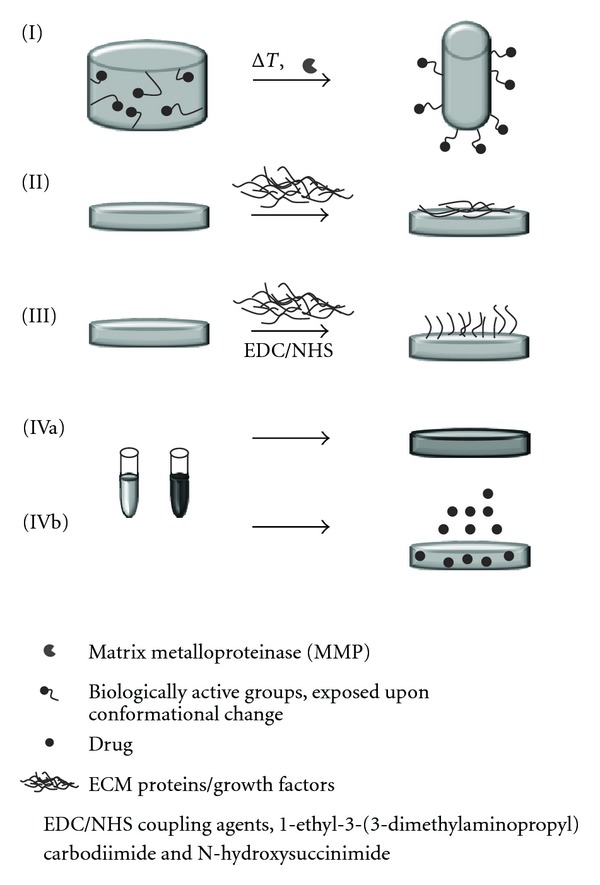
Schematic illustration of different functionalization principles. (I) Smart materials, changing conformation, and exposing different chemical groups upon temperature change or a change in enzyme concentration. (II) Surface functionalized materials. A synthetic scaffold is immersed in a ECM protein solution, allowing for protein adsortion on the surface. (III) Covalently functionalized scaffolds. Functional proteins are coupled to the surface via EDC/NHS chemistry. (IV) Blend materials, (a) hybrid scaffolds of various polymers or (b) hybrid scaffolds of polymers and drugs for controlled release.

In the future, the potential of engineered tissue and in particular the scaffold type as well as better understanding of biomaterial-cell interactions are of paramount importance toward successful cardiac regeneration. Initiated with only a few mandatory factors such as being biocompatible, non-cytotoxic, and providing a three-dimensional framework for cells to attach and develop, scaffolds for tissue engineering have evolved into more and more highly sophisticated and custom tailored constructs. Incorporating peptides, proteins, or growth factors renders an inert synthetic scaffold biologically active [45, 46] and facilitates regulation of cell expression via material properties and at the site of interest. Surface immobilized growth factors bypass the problems of rapid diffusion, short blood plasma half-life, and potential health risk as seen for soluble factors injected into the blood stream [47, 48]. Focusing on functionalization of cardiac constructs, four main approaches have been investigated so far: (I) smart materials, (II) surface modified materials via adsorption, (III) surface modified materials via covalent immobilization, and (IV) blended materials. Concepts of substrate functionalization are presented in Figure 2.

### 5.1. Smart Materials

Smart materials are materials that alter their shape, color, or size in response to an external stimulus. Such an external stimulus can be a change in temperature, pH, electrical or magnetic field, light, or naturally occurring enzymes. In the field of tissue engineering, materials showing smart behavior are mainly, if not exclusively, restricted to hydrogels showing thermo- or enzyme-responsive behavior. As early as the sixties, Wichterle and Lim [49] characterized a hydrophilic gel for biological use. Although consisting up to 99% of water, hydrogels sustained their position over the years and gained increasing interest in tissue engineering and numerous reviews summarize the concept of bioresponsive hydrogels for tissue engineering or drug delivery [50–55]. The concepts of stimuli-dependent conformational changes of polymers have only recently bridged from chemical laboratories and theoretical application to *in vitro *and *in vivo *studies.

#### 5.1.1. Thermosensitive Hydrogels

Thermosensitive hydrogels are mostly based on poly(N-isopropylacrylamide) (pNI-PAAm). Upon cooling from 37°C to 32°C, the polymer switches from a hydrophobic to a hydrophilic state. The previous state allows for cell attachment, whereas the latter one causes cell sheet release from the substrate. For a detailed description of the mechanism, see Graziano as well as Baysal and Karasz [56, 57]. Shimizu et al. [58] cultured neonatal rat cardiomyocytes on temperature-sensitive pNI-PAAm-coated dishes. Cell sheets were detached and overlaid to construct a four-layered cardiac graft. Studies of subcutaneous implantation in rats showed constant beating and vascularization of the construct. Following the same principle, Kubo et al. [59] cultured neonatal rat cardiomyocytes on pNI-PPAm to design myocardial tubes of wrapped cell sheets. In a combined study of thermo-sensitive and micropatterned pNI-PAAm-ECM films, the creation of multilayered oriented constructs of cardiomyocytes and C2C12 myoblasts was shown [60]. These studies demonstrated promising results for the *in vitro *preconditioning of cell cultures and, subsequently, scaffold-free implantation. This concept is one of the first to reach clinical trials.

#### 5.1.2. Enzyme-Sensitive Hydrogels

Enzyme or more specifically, matrix metalloproteinase- (MMP-) sensitive hydrogels always consist of two parts: a MMP-sensitive component (generally an ECM protein) and a component that controls changes in (non)covalent interactions (a synthetic polymer) that then cause macroscopic transitions. Excellent articles by Lutolf et al. [55] and Ulijn [50] describe the underlying principles and mechanisms. Most MMP-sensitive hydrogels provide sites for disease-specific enzymes that degrade the hydrogel, allowing either cell invasion or drug release and represent the most prominent candidates for application in tissue engineering. Indicating the importance of degradable hydrogels, Shapira et al. [61] conducted a study of neonatal rat cardiomyocytes on a MMP-sensitive PEGylated fibrinogen hydrogel. A different cell morphology was induced in MMP-2- and MMP-9-deficient cell cultures compared to control cultures with MMP. Other experimental studies focused on the functionalization of hydrogels with cell adhesion rather than MMP-sensitive motives. Yu and coworkers[62] designed an RGD-modified alginate hydrogel and could show increased proliferation of human umbilical vein cord endothelial cells (HUVECs) and increased angiogenesis in a rat infarct model. Combining cell adhesion motives, enzyme-sensitive scaffolds and drug release in one construct, Phelps et al. [63] engineered PEG-based bioartificial hydrogel matrices presenting MMP-degradable sites as growth factor release system, RGD peptide as cell adhesion motifs, and VEGF to induce the growth of vasculature *in vivo*. They reported that implantation of their construct induced the growth of new vessels into the matrix *in vivo *and resulted in significantly increased rate of reperfusion in a rat limb ischemic model.

### 5.2. Surface-Modified Materials via Protein Adsorbance

Surface-modified materials are among the most frequently functionalized materials. Synthetic, biologically inert polymer scaffolds are rendered bioactive by a coating of naturally occurring ECM proteins. The manifold techniques and approaches of hybrid scaffolds of naturally occurring and synthetic polymers are summarized in reviews by Furth et al. and Rosso et al. and particularly focused on cardiac tissue engineering, in Chan et al. [64–66]. Interestingly, in a comparative study of fibronectin-, collagen-, or laminin-coated elastomer poly(1,8-octanediol-co-citric acid) (POC), Hidalgo-Bastida et al. [67] could demonstrate most promoted cell adhesion of HL-1 mouse cardiac muscle cells on fibronectin-coated substrates. In a completely different study, C2C12 mouse myoblasts were shown to align and differentiate into myotubes on collagen-coated electrospun scaffolds of DegraPol [68]. Following the concept of contact guidance, McDevitt et al. [69] constructed micropatterned laminin lanes on poly(dimethylsiloxane) (PDMS) substrates to promote cell alignment of cardiomyocytes. Several years later, Cimetta et al. [70] produced contractile cardiac myografts on laminin-coated (microprinted) poly(acrylamide) hydrogels. Easy setup and high reproducibility made the setup a potential candidate for application in high-throughput biological and physiological studies. The straightforward method of protein adsorbance, however, carries several drawbacks such as uncontrolled release and unknown conformation of the protein on the surface. Furthermore, adsorbed protein concentration can only be inaccurately controlled. Alternatively, the concept of covalently linked proteins arose.

### 5.3. Surface-Modified Materials via Covalent Immobilization

Growth factors play an essential role in tissue engineering, and ideally, they would not be supplied in a soluble, quickly degradable form but be active at the site of interest, that is, at the scaffold surface and in the targeted host tissue. Newly developed materials influence the neovascularization processes in ischemic tissue since they allow the delivery of vascular endothelial growth factor (VEGF) and basic fibroblast growth factor (FGF), two main factors that control neoangiogenesis. Immobilized VEGF has been confirmed [47, 48, 71] to induce extended signaling and enhanced biological activity compared to soluble VEGF, as shown by increased proliferation of endothelial cells (ECs). Shen et al. [72] furthermore demonstrated increased cell infiltration of endothelial cells into a VEGF-functionalized scaffold, compared to cell cultures where VEGF is supplied in soluble form in the medium. In an advanced study, Chiu et al. [73] covalently immobilized VEGF and angiopoietin-1 (Ang-1) on a porous collagen scaffold, resulting in enhanced vascularization in a CAM assay and improved tube formation by endothelial cells. Furthermore, gelatin has been grafted to air plasma-activated PCL scaffolds via coupling agents such as 1-ethyl-3-(3-dimethylaminopropyl)carbodiimide and N-Hydroxysuccinimid (EDC/NHS coupling chemistry). The modified substrate enhanced spreading and proliferation of endothelial cells. Additionally, endothelial cells followed the fibre orientation of gelatin-coated scaffolds as compared to pure PCL scaffolds where a random cell orientation was found [74]. Although used in many approaches, immobilizing proteins or growth factors, via EDC/NHS chemistry, promote several issues. EDC/NHS coupling generates highly heterogeneous structures, regarding function and orientation of the immobilized proteins. Backer et al. [47] developed a new, site-specific covalent immobilization approach. A genetically induced N-terminal Cys-tag of VEGF coupled the growth factor to fibronectin. Controlled orientation was achieved. VEGF receptors were stimulated by immobilized VEGF and are fully capable of signal transduction pathways. Rather simple chemistry confirmed biological activity, increased endothelial cell proliferation, and reported that vascularization in a CAM model render VEGF-functionalized scaffolds a promising substrate for *in vivo* vascularization of ischemic myocardium. However, *in vivo* studies of functionalized substrates are still in their infancy. Experimental studies by Banfi and coworkers [75–77] emphasized the critical role of microenvironmental VEGF concentration. Timing of the expression, concentration gradient, and interactions with cells are all critical issues that need to be taken into account when designing VEGF scaffolds. A critical threshold defines both normal and aberrant angiogenesis. In summary, the spatiotemporal distribution of VEGF to stimulate the formation of stable new vessels in a scaffold must be addressed and investigated. Studies on VEGF release from alginate/chitosan hydrogel were performed by De laRi Va et al. [78], indicating a first burst effect, followed by constant release over 5 weeks. For cardiac implants, we, however, aim for immobilized factors, stimulating the regeneration of ischemic tissue over a longer period of time.

In 2003, a clinical study on the safety and efficacy of intracoronary and intravenous infusion of rhVEGF was conducted [79]. In the so-called VIVA trial (vascular endothelial growth factor in ischemia for vascular angiogenesis), 178 patients with stable exertional angina were randomized to receive placebo, low-dose (17 ng kg^−1^ min^−1^), and high-dose (50 ng) rhVEGF by intracoronary infusion, followed by intravenous infusion on days 3, 6, and 9. VEGF was safe and well tolerated; high-dose VEGF resulted in significant improvement in angina after 120 days. Despite promising results, this was only a small trial with a short-term followup.

### 5.4. Blend Materials and Drug Release

A straightforward technique that potentially involves all aforementioned concepts of functionalization constitutes the production of material blends. Following the same rationale of optimizing the bioactivity of scaffolds, synthetic materials can be blended with naturally occurring extracellular matrix (ECM) proteins, growth factors, or simply other synthetic materials to alter mechanical properties. In a straightforward setup, Choi et al. [80] produced an electrospun substrate of aligned polycaprolactone/collagen fibres and could induce skeletal muscle differentiation and myotube formation thereon. Using a similar scaffold, Tillman et al. [81] confirmed the potential of polycaprolactone/collagen scaffold for *in vitro* cell culture of endothelial progenitor cells; furthermore, the construct was shown to retain its structural integrity over one month in a rabbit aortoiliac bypass model. The endothelialized substrates resisted blood platelet adherence in the animal model.

Synthetic or natural polymers can, however, not only be blended among each other but also drugs serve as essential supplements in material design for biomedical engineering. Kraehenbuehl et al. [82] developed a matrix metalloproteinase- (MMP-) degradable polyethylene glycol (PEG) construct with incorporated thymosin *β*4. Entrapped T*β*4 promoted enhanced endothelial cell survival, cadherine, and angiopoietin-2 expression and increased MMP-2 and MMP-9 production. Metalloproteinase production directly stimulated the hydrogel degradation and T*β*4 release, providing a controlled drug release system.

Interestingly, Thakur et al. [83] combined two different drugs in a poly(L-lactic acid) (PLLA) electrospun scaffold. lidocaine and mupirocin showed different release kinetics when incorporated into the fibrous mesh. The hybrid scaffold can be employed for wound dressing, where a fast release of Lidocaine promotes immediate pain relief, whereas a sustained release of Mupirocin provides a constant antibiotic function until wound healing. The dual-release profile concept can find wide application in tissue engineering, enabling scientists to develop spatiotemporal controlled drug release.

### 5.5. Nanoparticles and Drug Release

Furthermore, nanotechnologies offer the possibility to design nanoparticles for drug delivery with large possibilities for customization of the particles. In particular, functionalized surfaces allow targeting of the particle, and tailored solubility, size, and shape present advantages for drug encapsulation and optimized biodistribution [84]. The nanosized particles are especially designed for the delivery of drugs with intracellular targets. For instance, Dvir et al. recently targeted cardiac cells within the infarcted heart [85]. The authors designed nanoscaled liposomes, functionalised with an angiotensin II type I (AT1) receptor-specific peptide sequence. Twenty-four hours after injection, the particles were found mainly accumulated in the left ventricle of infarcted mice hearts.

A controlled drug release at the site of interest may be controlled by enzyme-, pH or temperature-sensitive hydrogel systems. Wang et al. [86] performed an intramyocardial administration of bFGF-loaded temperature-sensitive chitosan hydrogel and reported on an attenuated remodeling, reduced infarct size, and increased arteriole numbers.

Finally, using a multiapproached experimental design, Ye et al. [87] injected skeletal myoblasts, transfected with a hypoxia-regulated VEGF plasmid that was encapsulated in polyethylenimine nanoparticles, into normal and infarcted hearts. The transfected myoblasts showed an improved cell survival and induced improved global LV function in a rabbit model of myocardial infarction.

## 6. Conclusion

Various strategies for cardiac diseases, including tissue engineering and stem-cell-based therapy, have been investigated in the past decade. Solving challenges that have arisen is a pressing objective for cardiac reparative medicine. Nevertheless, we can realistically predict that future treatments will include cell-based therapies. However, so far only short-to mid-term results have been provided. The long-term evaluation of possible heart recoveries remains to be confirmed, in particular for engineered tissue using rapidly degradable scaffolds. Controls of cell matrix interaction, dose, and time delivery will represent major breakthroughs for refining treatment and will govern successful clinical applications.

## Figures and Tables

**Table 1:** Potential cell source.

Source	Definition	Drawbacks
	Donor/recipient	
Autologous	Same individual	Not always available (genetic diseases, age)
Allogenic	Same species	Immunological issues
Xenogenic	Different species	Ethical issues and rejection
Syngenic or isogenic	Genetically identical individuals (clones, inbred)	Most appropriate for research with animal model

Origin/differentiation		

Primary	Tissue or organ/specialized	Large expansion needed
Secondary	Cell bank	Cryopreservation/immunological issues
Embryonic stem cells (iPSCs)	Undifferentiated	Ethical issues/purification/teratoma
Adult stem cells	Commited	Selection of type/source

**Table 2:** Potential cells for new therapeutic treatment.

Candidates	Concerns	Side effects	Mechanism of action	Clinical trials	Change in cardiac function (% EF versus ctrl.)*
Human embryonic stem cells	Ethics purification	Teratoma	Differentiation/myogenesis	FDA approval	
Fetal/neonatal cardiac muscle cells	Ethics accessibility		Differentiation/myogenesis	×	
Induced pluripotent stem cells		Teratoma	Differentiation/myogenesis	×	
Cardiac stem cells			Differentiation/myogenesis	*✓* (2009)	−0.2; +6.0
Skeletal muscle myoblasts	Poor electrocoupling	Arrhythmia	Paracrine effect	*✓*	+3; +14
Bone marrow stem cells	Purification/loss of function with age	Arrhythmia?	Paracrine effect	*✓*	−3.0; +12
Progenitors	Survival and controlled differentiation		Paracrine effect	*✓*	+2.8, +6.3

*EF: ejection fraction of the treated heart compared to control groups (adapted from Segers and Lee, 2008 [28]).
